# Thermally Developing Flow and Heat Transfer in Elliptical Minichannels with Constant Wall Temperature

**DOI:** 10.3390/mi10100713

**Published:** 2019-10-21

**Authors:** Liangbin Su, Zhipeng Duan, Boshu He, Hao Ma, Zairan Xu

**Affiliations:** School of Mechanical, Electronic and Control Engineering, Beijing Jiaotong University, Beijing 100044, China; 16116368@bjtu.edu.cn (L.S.); 18116018@bjtu.edu.cn (H.M.); 16222053@bjtu.edu.cn (Z.X.)

**Keywords:** elliptical minichannel, Nusselt number, thermal entrance length, constant wall temperature

## Abstract

Laminar convective heat transfer of elliptical minichannels is investigated for hydrodynamically fully developed but thermal developing flow with no-slip condition. A three-dimensional numerical model is developed in different elliptical geometries with the aspect ratio varying from 0.2 to 1. The effect of Reynolds number (25 ≤ *Re* ≤ 2000) on the local Nusselt number is examined in detail. The results indicate that the local Nusselt number is a decreasing function of Reynolds number and it is sensitive to Reynolds number especially for *Re* less than 250. The effect of aspect ratio on local Nusselt number is small when compared with the effect of Reynolds number on local Nusselt number. The local Nusselt number is independent of cross-section geometry at the inlet. The maximum effect of aspect ratio on local Nusselt number arises at the transition section rather than the fully developed region. However, the non-dimensional thermal entrance length is a monotonic decreasing concave function of aspect ratio but a weak function of Reynolds number. Correlations for the local Nusselt number and the thermal developing length for elliptical channels are developed with good accuracy, which may provide guidance for design and optimization of elliptical minichannel heat sinks.

## 1. Introduction

Laminar convective heat transfer in mini- and micro-channel heat sinks has emerged as a significant research area in recent years. This has been motivated by the demand for the highly effective heat removal property in various applications, such as aerospace, electronic chips and medical and biomedical use. Microchannels are a fundamental part of microfluidic systems. A number of researches have been conducted to understand the mechanism of fluid flow and heat transfer characteristics in microchannels with circular [1,2,3], parallel plates [4,5,6], annular [7,8,9], rectangular [10,11,12,13,14,15] and trapezoidal [16,17,18] cross-sections. Compared with a circular duct, a better thermal efficiency can be found in an elliptical passage because the perimeter of an elliptical configuration is wider than the other for the same cross-sectional area [19]. Generally, the elliptical channel heat sinks design can significantly reduce the size and weight of the apparatus [20]. Furthermore, it is worth noting that the elliptical shape is very common in current mini- and micro-devices since it can be easily manufactured with micro-drilling techniques or chemical etching. It is thus indispensable to determine the fluid flow and heat transfer performance in elliptical ducts.

Shah and London [21] summarized some work about the fully developed flow and heat transfer in elliptical ducts. They defined several kinds of thermal boundary conditions, such as *T* boundary (constant wall temperature peripherally as well as axially), H1 boundary (constant axial wall heat flux with constant peripheral wall temperature) and H2 boundary (constant axial wall heat flux with uniform peripheral wall heat flux). *T* boundary condition is available for condensers, evaporators and automotive radiators (at high flows), where the boiling point and the freezing point are holding constant values to meet the demand. Abdel-Wahed et al. [22] experimentally studied the laminar developing flow and heat transfer inside an elliptical duct having an aspect ratio of 0.5. They obtained the velocity profiles at the entrance region and found the complete velocity development in the elliptical duct is achieved earlier in the minor axis than the major axis. Similar results were found by Velusamy and Garg [23], who investigated the laminar flow in the entrance region of elliptical ducts using a control volume-based solution. They found the relative growth rate of boundary layer thickness along the major axis of the duct is slower than that along the minor axis. They also observed that the isoaxial-velocity contours are not concentric ellipses. For the convective heat transfer in the thermal developing flow of elliptical ducts, Sakalis et al [24] analyzed numerically the effect of aspect ratio on the Nusselt number with three thermal boundary conditions, that are, constant temperature, circumferential uniform and axially linearly or exponentially varying temperature. Their results showed that the peak value of the fully developed Nusselt number can be found when the aspect ratio is 0.5 under the constant wall temperature boundary condition. They further investigated the laminar flow in a curved duct of elliptical cross-section with fins and found the heat transfer rate is the maximum with the same aspect ratio [25]. However, Shariat et al [26] numerically calculated the Nusselt number of laminar mixed convection in elliptic channels with a constant heat flux boundary condition, and found that the best heat transfer performance appears when the aspect ratio is 0.75. The difference may be ascribed to the different thermal boundary conditions and the nanofluid chosen as the working fluid. In addition, Ragueb and Mansouri [27] numerically examined laminar heat transfer of non-Newtonian fluid in elliptical ducts by taking into account the viscous dissipation with constant wall temperature. The results indicated that the value of the fully developed Nusselt number increases with the aspect ratio. They further studied the laminar forced convection of non-Newtonian nanofluids in elliptical ducts using the generalized integral transform technique under the same wall boundary condition [20]. Their results demonstrated that the heat transfer coefficient can be improved dramatically when reducing aspect ratio and increasing nanoparticle concentration. The same method had been adopted by Maia et al. [28] to obtain the temperature distribution and the convective heat transfer coefficient of non-Newtonian fluids at the entrance region in elliptical ducts. They found that the aspect ratio of the elliptical section has a marked influence on the heat transfer parameters and the peak value of Nusselt number arises as the aspect ratio is near 0.3, which agrees well with the results in Shah and London [21].

Flow and heat transfer characteristics of elliptical microchannels with slip/jump boundary conditions have also been studied. Duan and Muzychka [29] analytically examined the slip flow in elliptic microchannels using the separation of variables in elliptic cylinder coordinates. A simple model of the Poiseuille number in elliptic microchannels for slip flow was proposed, which can be used to predict mass flow rate and pressure distribution of slip flow in elliptic microchannels. Vocale et al. [30,31] further numerically investigated the heat transfer of fully developed flow in elliptic microchannels with velocity-slip and temperature-jump boundary conditions. The results showed that Nusselt number increases strongly with the aspect ratio for elliptical ducts under constant heat flux boundary. A semi-analytic Ritz method was utilized by Wang [32] to treat slip flow in arbitrarily curved microchannels. The results showed that surface slip of a curved duct not only promoted the flow rate, but also shifted the maximum velocity towards the outer boundary and the minimum velocity towards the inner boundary.

However, the effect of Reynolds number on Nusselt number is often ignored. Some work about the effect has been done for simultaneously developing flow [33]. While, for thermal developing flow, to the authors’ best knowledge, there is little work about the local Nusselt number and thermal entrance length varying with Reynolds number in elliptical minichannels, which are focused on in this paper. In addition, modeling gas microflows requires us to take into account the mean molecular spacing and the mean free path, which affects the Knudsen number *Kn* [34,35,36]. For *Kn* < 0.001, the flow can be assumed as a continuum flow and it is accurately modeled by the Navier–Stokes and energy equations with no-slip and no-temperature jump conditions at the walls. In this study, Knudsen number is far below 0.001. The thermal developing flow through elliptical minichannels is thus analyzed under the continuum assumption. The primary goal of the present paper is to analyze the effects of Reynolds number, ranging from 25 to 2000, and aspect ratio, ranging from 0.2 to 1, on the forced convective heat transfer in the entrance region of elliptical minichannels. A computational fluid dynamics (CFD) method is employed to obtain the temperature fields. The thermal entrance length and Nusselt numbers are thus deduced. Finally, the empirical correlations for the heat transfer coefficient and thermal entrance length of thermal developing gas flow under constant wall temperature are developed to provide guidance for thermal design and optimization of elliptical minichannels.

## 2. Mathematical Formulations 

**Figure 1:**
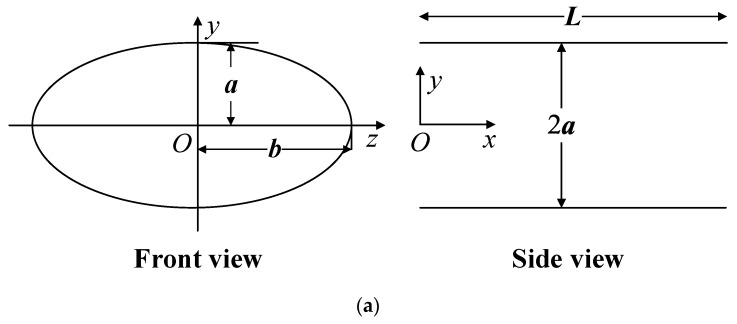

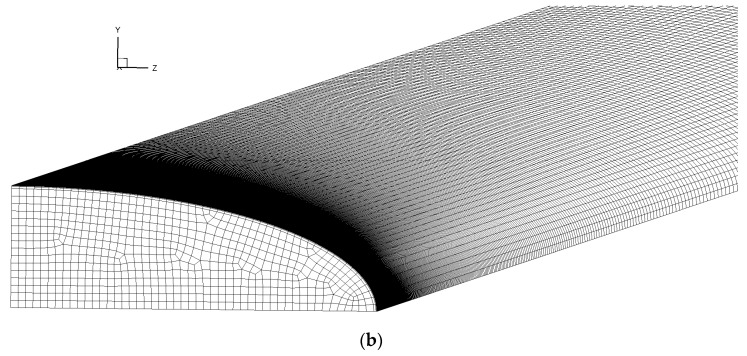
(**a**) Schematics of an elliptical minichannel; (**b**) numerical mesh of the quarter-channel computational domain.

A schematic diagram of a typical elliptical minichannel with coordinates and other dimensional nomenclature is shown in Figure 1a. The axial flow is in the *x* direction. Since the velocity and temperature fields are symmetric with respect to the *x*-*y* and *x*-*z* planes, a quarter of the geometry is chosen as the numerical domain to reduce computational costs, as shown in Figure 1b. In the present work, the hydraulic diameter *D*_h_ of the channel is calculated as

(1) $$D_{h}=\frac{4A}{P}=\frac{\pi a}{E(e)}$$

where *A* and *P* are the cross-section area and the wetted perimeter of channels respectively, *a* is the minor semi-axis and *E*(*e*) is the complete elliptical integral of the second kind. The eccentricity *e* of the ellipse is defined as:

(2) $$e=\sqrt{1-\varepsilon^{2}}$$

where the aspect ratio *ε* of an elliptical channel is defined as

(3) $$\varepsilon =\frac{a}{b}$$

where *b* is the major semi-axis.

In the present work, *D*_h_ is held constant at 200 μm in the numerical model and the axial length *L* is long enough to guarantee that the temperature profiles are fully developed before the channel exit. Therefore, *b* and *a* are varied with aspect ratio.

For thermal developing flow, the velocity profile is fully developed and the distribution at each cross-section in an elliptical duct is given in Shah and London [21] as:

(4) $$u(y,z)=2u_{m}\left(1-\frac{y^{2}}{a^{2}}-\frac{z^{2}}{b^{2}}\right)$$

where *u*_m_ is the flow average velocity and varies with the Reynolds number *Re*, namely,

(5) $$u_{m}=\frac{\mu Re}{\rho D_{h}}$$

where *μ* is dynamic viscosity and *ρ* is density. The following assumptions are applied in the present simulations, that are: steady-state; incompressible; laminar flow; negligible axial conduction and viscous dissipation; and constant fluid properties. The energy equation according to the above hypotheses is given as

(6) $$\rho c_{p}u\frac{\partial T(x,y,z)}{\partial x}=\lambda \left[\frac{\partial^{2}T(x,y,z)}{\partial y^{2}}+\frac{\partial^{2}T(x,y,z)}{\partial z^{2}}\right]$$

where *λ*, *c*_p_ are thermal conductivity and specific heat respectively. The Prandtl number of the fluid in the present work is specified as unity, correspondingly, *μ* = 2.4 × 10^−5^ kg/(m·s), *ρ* = 1.225 kg/m^3^, *λ* = 0.0242 W/(m·K) and *c*_p_ = 1006 J/(kg·K). The energy equation is non-dimensionalized when introducing the following dimensionless parameters:

(7) $$Y=\frac{y}{D_{h}};\;Z=\frac{z}{D_{h}};\;X=\frac{x}{D_{h}RePr};\;U=\frac{u}{u_{m}};\;\theta =\frac{T_{w}-T}{T_{w}-T_{\mathrm{in}}}$$

$$\alpha =\frac{a}{D_{h}};\;\beta =\frac{b}{D_{h}}$$

where the *T*_w_ is the temperature of the wall and *T*_in_ is the inlet temperature of the fluid. Equation (6) is thus transformed into the following dimensionless form:

(8) $$U\frac{\partial \theta }{\partial X}=\frac{\partial^{2}\theta }{\partial Y^{2}}+\frac{\partial^{2}\theta }{\partial Z^{2}}$$

The boundary conditions can be written with the dimensionless form. For the wall boundary, no-slip and no-temperature jump boundary conditions are set at the wall:

(9) θ=0

For inlet boundary, a uniform temperature profile is given, that is:

(10) θ=1

For *x*-*z* plane boundary, the heat flux is zero, that is:

(11) $$\frac{\partial \theta }{\partial Y}=0$$

The same condition for *x*-*y* plane boundary:

(12) $$\frac{\partial \theta }{\partial Z}=0$$

The zero pressure gradient is employed at the outlet.

Based on the temperature distribution of the fluid obtained by numerical simulation, the local surface heat flux $q^{\prime \prime }(x,y,z)$ at the wall is defined as

(13) $$q^{\prime \prime }(x,y,z)=\lambda \frac{\partial T}{\partial \vec{n}}$$

The direction of the local heat flux is the normal direction of the corresponding wall. Then the local convective heat transfer coefficient *h*(*x*) can be determined by the following equation,

(14) $$h(x)=\frac{q^{\prime \prime }(x)}{T_{w}-T_{m}(x)}$$

where *T*_m_(*x*) is the mass-weighted average temperature of the fluid in the cross-section and calculated with the following equation:

(15) $$T_{m}(x)=\frac{1}{A}\int_{\Omega }TUd\Omega =T_{w}-\frac{T_{w}-T_{\mathrm{in}}}{\pi \alpha \beta }\int_{\Omega_{X,Y}}\theta Ud\Omega_{X,Y}$$

$q^{\prime \prime }(x)$ is the mean heat flux at each cross-section, namely,

(16) $$q^{\prime \prime }(x)=\frac{1}{P}\int_{\Gamma }q^{\prime \prime }(x,y,z)d\Gamma$$

The local Nusselt number *Nu*(*x*) is obtained as

(17) $$Nu(x)=\frac{h(x)\cdot D_{h}}{\lambda }$$

In dimensionless form, it can be written as

(18) $$Nu(x)=\frac{-\int_{\Gamma }\frac{\partial \theta }{\partial n}d\Gamma }{4\int_{\Omega_{X,Y}}\theta Ud\Omega_{X,Y}}$$

### 2.1. Solution Method

With the rapid development of numerical algorithms, numerical simulation is becoming a more and more promising method to obtain detailed and approximate solutions, especially at the entrance region of some noncircular ducts where theoretical analytical solutions are quite complex. In the present work, a CFD software Fluent 6.3 (ANSYS, Inc., Canonsburg, PA, USA) was used to solve the flow and energy equations. The constant temperature of 300 K is imposed on the heated wall and the uniform inlet temperature of the fluid is 290 K. The small temperature difference makes the assumption of constant fluid properties realistic. To meet this demand of hydrodynamically developed flow at the inlet, there are three methods: The first is to lengthen the duct and ensure the fluid achieves fully developed condition before the specified inlet [27], the second is to embed a user-defined function and the third is to employ the profile function of the software [37]. There exists unnecessary numerical domain for the first method, which increases extra computational costs. For the second method, it is hard to present the velocity profile precisely. Considering the precision and computational costs, the third approach is adopted in the present work and a more detailed introduction about the profile function is shown in the User’s Guide [38]. The SIMPLE algorithm is chosen to deal with the coupling between pressure and velocity. The second-order upwind scheme is imposed on the governing equations. The convergence criterion for the residuals of the continuity and momentum equations are less than 1 × 10^−9^, and for energy equation is less than 1 × 10^−15^.

### 2.2. Grid Independence

**Figure 2:**
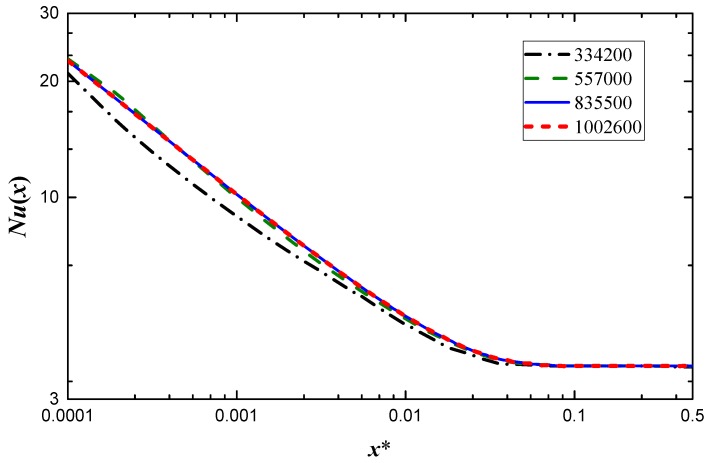
Nusselt number for four different grids.

The thermal boundary layer is quite an important area for the thermal developing flow where tremendous wall temperature gradient exists. The boundary layer mesh is thus adopted and the mesh in the near inlet region needs to be refined. The computational domain is meshed with hexahedral elements and a typical mesh is shown in Figure 1b. Several appropriate grids that are a suitable compromise between desired accuracy and solution cost need to be designed. The grid independence study is performed on a circular duct with *Re* = 2000 and four mesh sizes are considered, that are: (I) 334,200, (II) 557,000, (III) 835,500 and (IV) 1,002,600 grid cells. The effect of the grid size on Nusselt number is shown in Figure 2, where the abscissa x* is the non-dimensional axial coordinate and defined as

(19) $$x*=\frac{x}{D_{h}RePr}$$

where *Pr* is the Prandtl number. The III and IV mesh systems produce a variation of less than 0.26%. A minimum grid size of 835,500 is thus used for the study. The meshes of other conditions have also been tested and the grid size increases as the aspect ratio decreases due to the high curvature that exists at the end of major semi-axis.

### 2.3. Model Validation

The model validation is conducted in both fully developed region and thermal entrance region. For thermally fully developed flow in elliptical ducts, Shah and London [21] summarized some numerical values of Nusselt number under *T* condition. Maia et al. [28] analyzed the heat transfer of non-Newtonian fluids using the generalized integral transform technique with *T* boundary and the results of fully developed Nusselt numbers were shown with figures. The above basic data are used to validate the present numerical model in the fully developed condition and the results are shown in our previous work [33]. Good agreement can be observed and the deviations are less than 0.46%.

**Figure 3:**
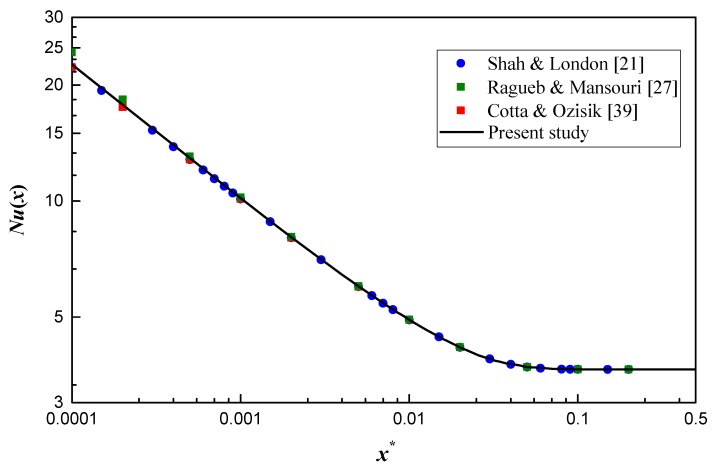
Validation of the numerical model for the thermal developing flow in a circular channel (*ε* = 1).

For thermal developing flow, there exists few available results on the elliptical channel. Shah and London [21] summarized numerical results of thermal developing flow in a circular duct. Ragueb and Mansouri [27] adopted a numerical method to calculate the thermal developing flow and heat transfer in elliptical channels and some data about a circular channel are listed in a table. Cotta and Ozisik [39] analytically studied the heat transfer at the thermal entrance region of non-Newtonian fluid inside a circular channel and the results of Newtonian fluid are shown with the tabular form. To validate the entrance problem in the present model, the present results are compared with the above data for a circular channel (a special case of elliptical ducts), as shown in Figure 3. A good agreement can be found and the maximum deviation is 7.1% when compared with the local Nusselt number in Ragueb and Mansouri [27]. The maximum deviation is reduced to 1.5% when compared with the results in Shah and London [21] and Cotta and Ozisik [39], which further verifies the model in the present work.

## 3. Results and Discussion

### 3.1. Local Nusselt Number

**Figure 4:**
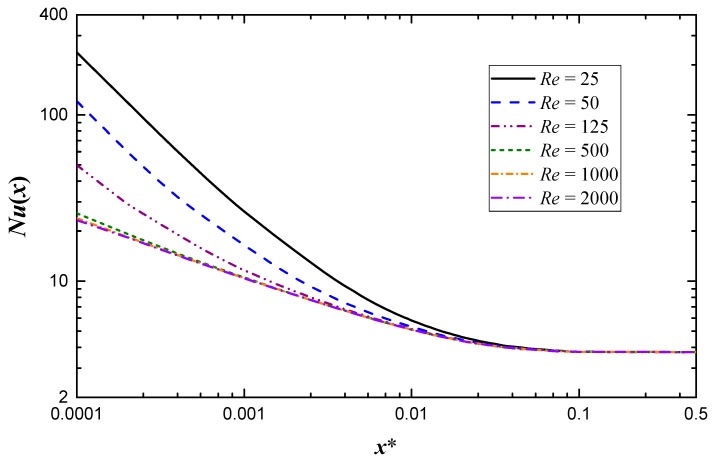
Local Nusselt number variation along the length of the channel with different Reynolds numbers for *ε* = 0.5.

The present work aims to investigate the effects of Reynolds number and aspect ratio on the heat transfer coefficient. Note that the effect of Reynolds number on local Nusselt number is often ignored. However, it needs to be considered when close to the inlet of channels [16]. The Nusselt number variation along the length of the channel with different Reynolds numbers for *ε* = 0.5 is shown in Figure 4. It is noticeable from the figure that *Nu*(*x*) is quite high at the entrance region and then decreases monotonically to approach the fully developed value. The effect of Reynolds number is apparent near the inlet and the effect gradually diminishes as x* increases. Generally, the minichannel is short enough and the entrance region needs to be considered, which makes the effect of Reynolds number cannot be ignored in minichannels. Besides this, the local Nusselt number is a decreasing function of *Re* at the same x*, that is, the local Nusselt number is higher when *Re* is smaller, which is contrary to the conventional conclusion. The interesting result can be ascribed to the non-dimensional abscissa. According to Equation (19), the practical location is closer to the inlet as *Re* decreases at the same x*.

**Figure 5:**
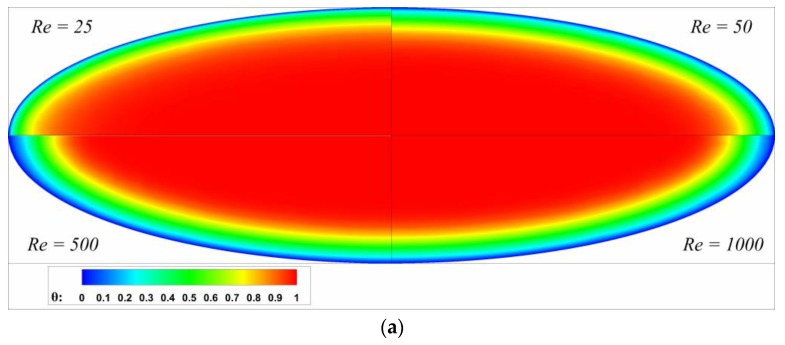

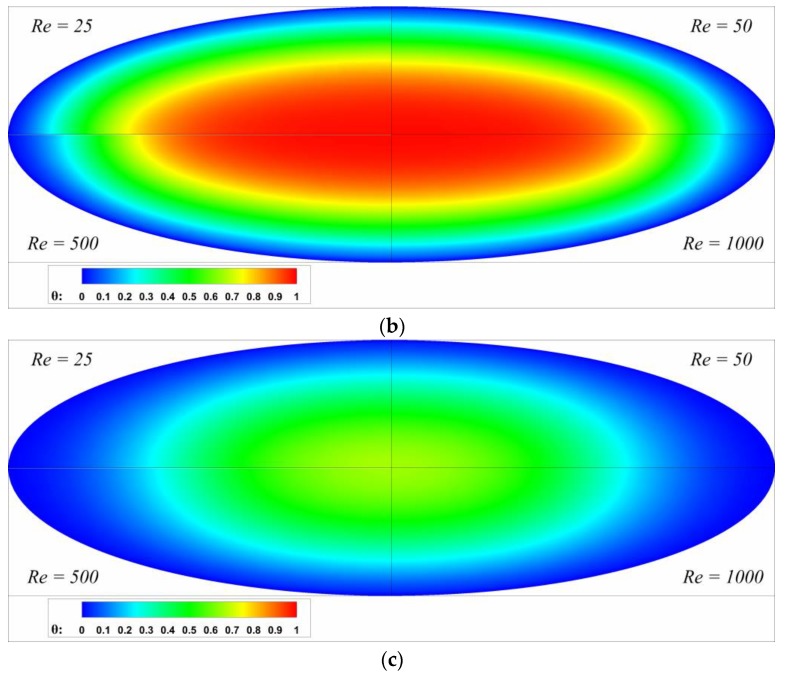
Dimensionless temperature profiles of different Reynolds numbers for *ε* = 0.33 at (**a**) x* = 0.001, (**b**) x* = 0.01 and (**c**) x* = 0.05.

The dimensionless temperature profiles with different *Re* are shown in Figure 5. The influence of *Re* on temperature distribution is obvious at the near inlet region, as shown in Figure 5a,b, and the wall temperature gradient is stronger for lower *Re*, which results in higher heat flux and larger Nusselt number. Nevertheless, the temperature distributions of different *Re* are nearly the same when close to the fully developed region, as shown in Figure 5c, which is in agreement with the results in Figure 4. In addition, there is no pronounced difference for the temperature distribution between *Re* = 500 and 1000 from x* = 0.001 to x* = 0.05, which indicates the effect of Reynolds number on local Nusselt number is negligible when *Re* over 500. Although not shown here, a similar effect of Reynolds number on temperature distribution is also observed in other channel geometries.

**Figure 6:**
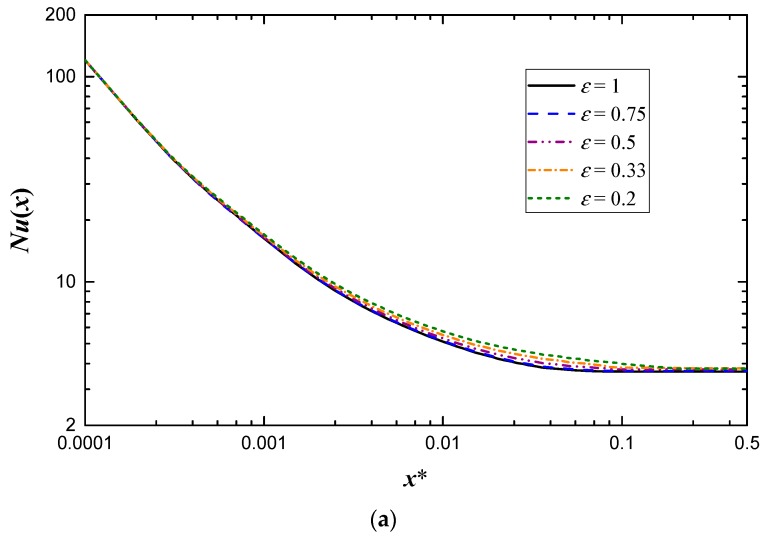

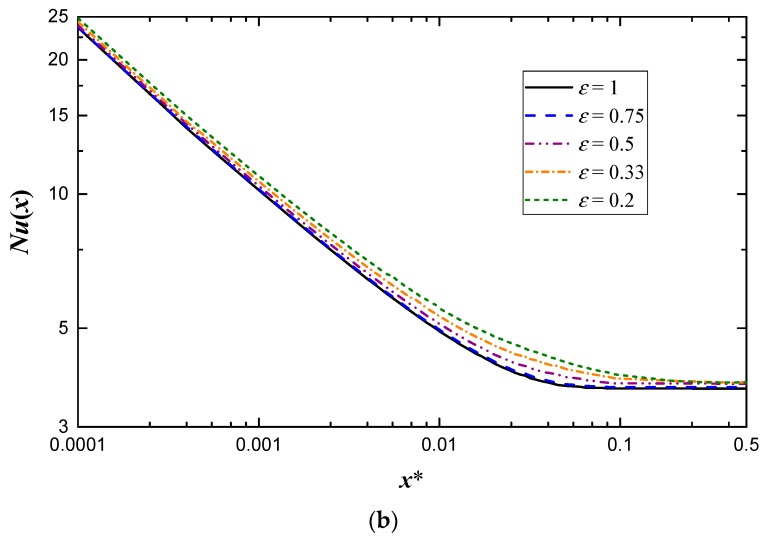
Local Nusselt number variation along length of channel with different aspect ratios at (**a**) *Re* = 50; (**b**) *Re* = 1000.

The effect of geometry on heat transfer performance is always a concern for researchers. Based on the present simulation, the effect of aspect ratio on the local Nusselt number is shown in Figure 6. It is obvious that the aspect ratio has little effect on local Nusselt number when close to the inlet as shown in Figure 6a, and the effect increases first and then decreases as x* increases. The maximum effect appears in the transition section where local Nusselt number decreases as aspect ratio increases. The same relationship between local Nusselt number and aspect ratio can be found at the entrance region, which is more apparent in Figure 6b. However, this dependence vanishes at the thermally fully developed region, where a peak value of Nusselt number emerges when the aspect ratio is near 0.33.

As displayed in Figure 6b, there is a distinct linear relation between *Nu*(*x*) and x* at the near inlet region, which is subject to the following type

(20) $${\left(Nu\right)}_{x*\to 0}=C{\left(x*\right)}^{n}$$

Similar results are found for *Re* = 500 and 2000. The index *n* determined by Reynolds number is the slope of the curve. *C* and *n* are obtained using the curve fitting method and the results are shown in Table 1. It is apparent that *n* decreases as *Re* increases, which is consistent with the results in Figure 4. *C* is a descending function of the aspect ratio but increases with Reynolds number. *Nu*(*x*) approaches the fully developed value when x* is greater than 0.2. The two linear relations on the logarithmic plot make it possible to adopt an asymptotic method [40] to build a model covering the whole range of x* as follows:

(21) $$Nu(x)≃{\{\left[{\left(Nu\right)}_{x*\to 0}\right]}^{N}+{{\left[{\left(Nu\right)}_{x*\to \infty }\right]}^{N}\}}^{1/N}$$

**Figure 7:**
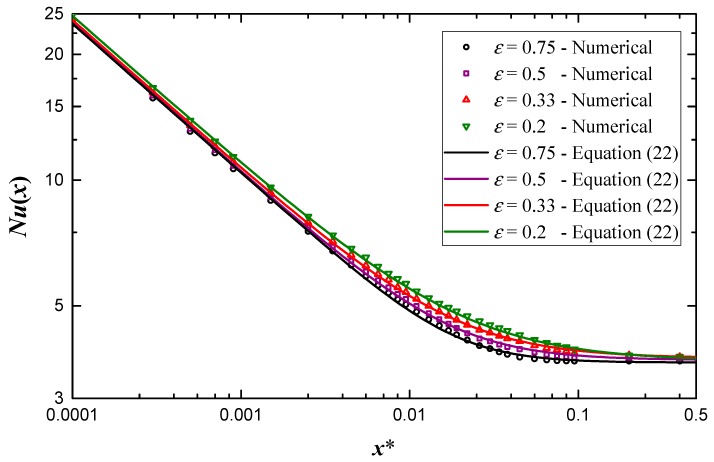
Comparison of Equation (22) and a portion of numerical results at *Re* = 1000.

In the present work, Equation (21) is further modified as follows to enhance the accuracy:

(22) $$Nu(x)≃{\{\left[{\left(Nu\right)}_{x*\to 0}\right]}^{N}+{{\left[{\left(Nu\right)}_{x*\to \infty }\right]}^{N-0.03}\}}^{1/N},\;\mathrm{for}\;0.0001<\;x*\;<\;0.5$$

where *N* is the blending coefficient and the values are listed in Table 1. Equation (22) predicts numerical data within ±3.1% when *Re* over 500, and the comparison of Equation (22) with a portion of numerical data is shown in Figure 7.

However, the linear relationship near the inlet region cannot be found when *Re* is less than 250, which makes it complicated to develop a correlation for the local Nusselt number. A sample of numerical data about *Nu*(*x*) at the entrance region for relative low *Re* is listed in Table 2 to provide a reference for future work.

### 3.2. Thermal Entrance Length

Based on the aforementioned results, it is clear that there is a high heat transfer efficiency at the entrance region because of the thin thermal boundary layer. A lot of research take steps to enhance heat transfer performance of heat sinks by redeveloping the boundary layer [41,42,43]. The thermal entrance length plays an important role in designing the heat exchanges. The thermal entry length *L*_th_ is defined as the axial length needed to achieve a value of *Nu*(*x*), which is 1.05 times the fully developed Nusselt number [44]. The non-dimensional thermal entrance length $L_{\mathrm{th}}^{*}$ is defined as

(23) $$L_{\mathrm{th}}^{*}=\frac{L_{\mathrm{th}}}{D_{h}RePr}$$

**Figure 8:**
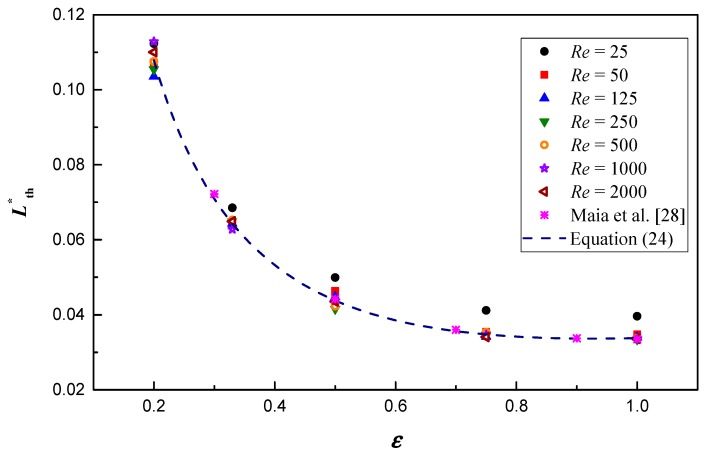
Dimensionless thermal entry length variation along the aspect ratio under different Reynolds numbers.

Figure 8 depicts the variation of the non-dimensional thermal entrance length. Clearly, $L_{\mathrm{th}}^{*}$ is a monotonic decreasing concave function of aspect ratio, which agrees well with the results of the Newtonian fluid in Maia et al. [28], and the data of Maia et al. [28] are delineated in Figure 8 to further verify the present work. Moreover, the effect of Reynolds number on $L_{\mathrm{th}}^{*}$ is small and irregular. When *Re* over 50, the effect can be neglected and a correlation is built by the curve fitting method to predict the present numerical data within ±5.4%, as follows

(24) $$L_{\mathrm{th}}^{*}=-0.1514+0.09621\ln\varepsilon +0.1852\varepsilon^{-0.5},\;\mathrm{for}\;50\;\leq \;Re\;\leq \;2000,\;0.2\;\leq \;\varepsilon \leq \;1$$

The correlation curve is shown in Figure 8 to make a contrast with the results in Maia et al. [28] and good agreements can be obtained, which further verifies the correlation.

## 4. Conclusions

In this paper, three-dimensional heat transfer of thermal developing flow in elliptical minichannels with constant wall temperature has been examined. The effects of Reynolds number (25 ≤ *Re* ≤ 2000) and aspect ratio (0.2 ≤ *ε* ≤ 1) on the local Nusselt number in both the entrance region and the fully developed region have been calculated numerically. The numerical model is first validated with the fully developed Nusselt number of elliptical ducts and further with the local Nusselt number of a circular channel. Good agreements are achieved between the present results and the published data.

Based on this investigation, it is found that the effect of Reynolds number on local Nusselt number cannot be neglected, which is attributed to the fact that the wall temperature gradient is sensitive to the Reynolds number especially when the Reynolds number is less than 250. For *Re* over 500, the apparent linear relationship between the local Nusselt number and non-dimensional channel length at near inlet region is found and a correlation is proposed to predict the results within ±3.1%. Compared with the effect of Reynolds number on local Nusselt number, the effect of aspect ratio is quite small at the entrance region, and the maximum effect of aspect ratio on local Nusselt number appears at the transition section rather than the fully developed region. The non-dimensional thermal entrance length is mainly influenced by aspect ratio, and a correlation is developed for the non-dimensional thermal entrance length in elliptical channels to predict the results within ±5.4%.

## Figures and Tables

**Table 1:** Curve-fit parameters for Equations (20) and (22).

Aspect Ratio	*Re* = 500		*Re* = 1000		*Re* = 2000	
	*C*	*N*	*C*	*N*	*C*	*N*
*ε* = 0.2	0.794	2.60	0.920	2.80	0.955	2.80
*ε* = 0.33	0.785	3.00	0.900	3.24	0.944	3.24
*ε* = 0.5	0.776	3.40	0.887	3.80	0.927	3.80
*ε* = 0.75	0.759	3.80	0.883	4.78	0.916	4.78
*ε* = 1	0.753	3.80	0.879	4.78	0.902	4.78
*n*	−0.379		−0.357		−0.350	

**Table 2:** A numerical sample of data for *Nu*(*x*).

*Re*	x*	*ε* = 0.2	*ε* = 0.33	*ε* = 0.5	*ε* = 0.75	*ε* = 1
25	0.0001	237.7	237.5	237.4	237.2	237.2
	0.0002	119.5	119.4	119.4	119.2	119.1
	0.0005	49.12	49.01	48.85	48.68	48.66
	0.001	26.77	26.57	26.24	26.19	26.05
	0.005	8.622	8.385	8.146	7.988	7.940
	0.01	6.256	6.017	5.803	5.645	5.604
50	0.0001	120.7	120.6	120.6	120.5	120.2
	0.0002	60.29	60.25	60.24	60.06	59.82
	0.0005	27.56	27.36	26.97	26.73	26.93
	0.001	17.02	16.73	16.46	16.30	16.29
	0.005	7.215	6.966	6.755	6.599	6.558
	0.01	5.742	5.512	5.306	5.152	5.114
125	0.0001	50.13	50.03	49.86	49.69	49.53
	0.0002	29.92	29.67	29.16	28.89	29.16
	0.0005	17.33	16.99	16.72	16.51	16.49
	0.001	12.18	11.87	11.63	11.43	11.40
	0.005	6.696	6.526	6.308	6.139	6.116
	0.01	5.578	5.353	5.148	4.985	4.958
250	0.0001	33.19	32.90	32.45	32.31	32.30
	0.0002	22.43	22.06	21.66	21.47	21.46
	0.0005	14.81	14.45	14.20	14.00	13.93
	0.001	11.22	11.00	10.76	10.58	10.53
	0.005	6.677	6.441	6.234	6.079	6.041
	0.01	5.550	5.325	5.121	4.968	4.931

## Nomenclature

*A*	flow area, m^2^
*a*	minor semi-axis of ellipse, m
*b*	major semi-axis of ellipse, m
*c*_p_	specific heat, J/(kg K)
*D*_h_	hydraulic diameter, m
*e*	eccentricity of the ellipse
*h*	convective heat transfer coefficient, W/(m^2^ K)
*Kn*	Knudsen number
*L*	channel length, m
*Nu*	Nusselt number, = *hD*_h_/*λ*
*P*	wetted perimeter, m
*p*	pressure, $N/m^{2}$
*Pr*	Prandtl number = *μc*_p_/λ
*q*’’	wall heat flux, W/m^2^
*Re*	Reynolds number, = $\bar{u}D_{h}/\nu$
*T*	temperature, K
*U*	non-dimensional velocity
*u*	velocity, m/s
*X*, *Y*, *Z*	non-dimensional Cartesian coordinates
*x*, *y*, *z*	Cartesian coordinates, m
*x**	dimensionless axial distance, = *x*/(*D*_h_*RePr*)
Greek symbols	
*α*	non-dimensional minor semi-axis of ellipse
*β*	non-dimensional major semi-axis of ellipse
*ε*	aspect ratio, = *a*/*b*
*θ*	dimensionless temperature
*λ*	fluid thermal conductivity, W/(m K)
*μ*	dynamic viscosity, $N\;s/m^{2}$
*ρ*	fluid density, kg/m^3^
Г	cross section contour
Ω	cross-section domain
Subscripts	
m	mean
th	thermal
w	wall
